# Supplementary material for: A high-resolution mRNA expression time course of embryonic development in zebrafish
Source: eLife. 2017 Nov 16;6:e30860. doi: 10.7554/eLife.30860 (PMC5690287; doi:10.7554/eLife.30860)
Supplement: Supplementary file 6. [file elife-30860-supp6.zip › biolayout-clusters-files/Cluster134-genes.html]

Cluster134


# Cluster134: Genes

| Ensembl ID | Gene Name | Chr | Start | End | Biotype |
| --- | --- | --- | --- | --- | --- |
| ENSDARG00000038141 | atf4b | 3 | 24067386 | 24070271 | protein\_coding |
| ENSDARG00000015050 | calm3a | 12 | 24833510 | 24838368 | protein\_coding |
| ENSDARG00000030326 | efna1a | 19 | 8562877 | 8583396 | protein\_coding |
| ENSDARG00000042670 | eps15l1a | 22 | 15881193 | 15930782 | protein\_coding |
| ENSDARG00000016089 | gltscr1l | 12 | 34775062 | 34786880 | protein\_coding |
| ENSDARG00000021647 | gnai1 | 4 | 22959467 | 22993088 | protein\_coding |
| ENSDARG00000025566 | slc25a3b | 25 | 3180945 | 3195621 | protein\_coding |
| ENSDARG00000092553 | slc25a5 | 14 | 32541311 | 32543824 | protein\_coding |
| ENSDARG00000031891 | tbx1 | 5 | 14702278 | 14715266 | protein\_coding |
| ENSDARG00000023323 | ywhaqb | 17 | 32547876 | 32573138 | protein\_coding |
